# The roles of lipids and inflammation in the association between the triglyceride-glucose index and arterial stiffness: evidence from two large population-based surveys

**DOI:** 10.1186/s12944-024-02183-0

**Published:** 2024-06-22

**Authors:** Jinlian Li, Pei Ye, Xiangyan Peng, Guangda Xiang

**Affiliations:** 1 The First School of Clinical Medicine, Southern Medical University, Guangzhou, Guangdong 510515 China; 2 Department of Endocrinology, General Hospital of Central Theater Command, Wuhan, Hubei 430070 China; 3 Shantou University Medical College, Shantou, Guangdong China

**Keywords:** Arterial stiffness, Triglyceride-glucose (TyG) index, Lipid, Inflammation, Mediation analyses

## Abstract

**Background:**

The triglyceride-glucose (TyG) index is a risk marker for arterial stiffness; however, the extent to which the TyG index is associated with arterial stiffness via lipids and inflammation remains unknown. The first aim was to probe the relationship between the TyG index and arterial stiffness in two surveys. The second aim was to clarify whether lipids and inflammation mediate this relationship.

**Methods:**

The sample size of 13,726 U.S. individuals from the National Examination Survey (NHANES) and 3,964 Chinese individuals from the China Health and Retirement Longitudinal Study (CHARLS 2015) were enrolled. Weighted multivariate logistic and linear regression models, as well as restricted cubic spline (RCS) and mediation analyses, were utilized to estimate complex relationships between the TyG index, arterial stiffness, lipids (non-high-density lipoprotein cholesterol [non-HDL-C]) and inflammation (C-reactive protein [CRP]) biomarkers.

**Results:**

A total of 3,420 U.S. patients and 992 Chinese patients were diagnosed with increased arterial stiffness. Regression analyses demonstrated that higher quartiles of the TyG index were associated with a greater incidence of increased arterial stiffness (NHANES: OR = 2.610, 95% CI = 2.043–3.334, *P* < 0.001; CHARLS: OR = 1.579, 95% CI = 1.057–2.360, *P* < 0.001). Participants with a higher TyG index/higher CRP level or with a higher TyG index/higher non-HDL-C level had the highest incidence of increased arterial stiffness in the two surveys. The results were still consistent when the sensitivity analysis was implemented with stricter clinical cut-off values of non-HDL-C. Mediation analysis verified that lipids (mediated effect: β = 0.012, *P* < 0.001 in NHANES; β = 0.020, *P* < 0.001 in CHARLS) and inflammation (mediated effect: β = 0.003, *P* < 0.001 in NHANES; β = 0.006, *P* < 0.001 in CHARLS) partially mediated this relationship.

**Conclusions:**

These results indicated a positive linear correlation between the TyG index, non-HDL-C level, CRP level and increased arterial stiffness in two surveys. Furthermore, lipids and inflammation could partly mediate the correlation of the TyG index with arterial stiffness in both surveys.

## Introduction

Arterial stiffness is closely correlated with cardiovascular disease (CVD) and its prognosis [1], which is still the main cause of death globally [2]. It is widely known that arterial stiffness is evaluated by using carotid-femoral pulse wave velocity (cfPWV), which is considered a reference measure [3]. Interestingly, emerging evidence has convincingly demonstrated that estimated pulse wave velocity (ePWV) is positively related to cfPWV [4] and strongly associated with vascular diseases [5]. For example, population-based researches have demonstrated that increased arterial stiffness assessed by ePWV is related to an increased risk of stroke, hypertension and CVD [6, 7].

Insulin resistance (IR) strongly increases the risk for arterial stiffness and CVD onset [8]. The euglycaemic-hyperinsulinaemic clamp was reported to be the “gold standard” for measuring IR [9]. Nevertheless, compared to the euglycaemic-hyperinsulinaemic clamp, the triglyceride-glucose (TyG) index is a reliable surrogate for IR [9]. Furthermore, the TyG index is economically efficient and more convenient for the assessment of arteriosclerosis [10]. More importantly, a recent study demonstrated that the TyG index outperforms the homeostasis model assessment for IR (HOMA-IR) in terms of IR evaluation, regardless of glycaemic status [11]. Indeed, the TyG index was shown to be more strongly linked to arterial stiffness and its progression than the HOMA-IR index in individuals who have hypertension or type 2 diabetes [12–14]. However, the precise mechanism mediating the relationship between the TyG index and arterial stiffness remains unclear.

Dyslipidaemia, especially hypercholesterolemia, can cause changes in vascular stiffness and is a risk factor of CVD [15]. Low-density lipoprotein (LDL) is currently the primary lipid-lowering target; however, its use is limited, particularly in patients with high triglycerides and diabetes [16]. Non-high-density lipoprotein cholesterol (non-HDL-C) constitutes all apoB-containing lipoprotein particles except for high-density lipoprotein cholesterol (HDL-C), which also includes triglyceride-rich remnants [15]. Numerous researches have indicated that non-HDL-C is a better predictor of CVD than LDL-C alone [17, 18]. Thus, we strived to probe the role of non-HDL rather than triglycerides and low-density lipoprotein.

Given that lipid (non-HDL-C) [19] and inflammatory (CRP) [20]biomarkers have been associated with both IR and arterial stiffness [21], recent reports have revealed a connection between arterial stiffness and IR. However, it remains to be elucidated as to whether lipid (non-HDL-C) and inflammation (CRP) levels could play important roles in the relationship between the TyG index and arterial stiffness. This study differs from previously published studies [14, 20, 21]. First, the study not only explored the complex associations of TyG index, non-HDL-C level and CRP level and arterial stiffness, but further explored whether lipid levels and inflammation status were key mediators of this relationship in both large surveys. Second, the combined valuation of insulin resistance, lipids and inflammation levels to further stratify the arterial stiffness risk was also recommended by this study. Moreover, to the best of our knowledge, no report has explored the mediating roles of lipid (non-HDL-C) and inflammation (CRP) levels in the relationship between the TyG index and arterial stiffness risk. Herein, the first aim of this study was to explore the correlation of the TyG index with arterial stiffness in two large investigations of the United States and Chinese populations, given that insulin resistance is a traditional predictor of arterial health. The secondary purpose was to test the hypothesis that lipids and inflammation partially mediate the correlation between the TyG index and arterial stiffness.

## Methods

### Study populations

The National Health and Nutrition Examination Survey (NHANES) is a national initiative of the National Center for Health Statistics (NCHS) that focuses on the nutritional and health conditions of the U.S. civilian population every two years, with the goal of providing a comprehensive understanding of the contemporary spectrum of disease to inform public health policy. Additionally, from a total of 101,316 baseline individuals in the NHANES (1999–2018), participants were excluded for the following reasons: (1) age < 20 years (*n* = 47,207); (2) lack of data on patients with diabetes and hypertension (*n* = 20,963); and (3) missing records of blood glucose, diastolic blood pressure (DBP), total cholesterol (TC), LDL-C, systolic blood pressure (SBP), and CRP (*n* = 19,420). The current study also obtained data from the China Health and Retirement Longitudinal Study (CHARLS 2015), which is a nationwide demographic cohort study of Chinese people aged over 45 years, with four regular surveys conducted every six months (http://charls.pku.edu.cn/). We selected the baseline individuals in the CHARLS (*n* = 21,097). The exclusion criteria for individuals were as follows: (1) < 45 years of age (*n* = 86), extreme BMI levels (> 55 or < 15 kg/m^2^) (*n* = 57), or DBP > SBP (*n* = 16); (2) lack of data on SBP and DBP (*n* = 4,689); and (3) lack of data on TC, uric acid (UA), HDL-C, LDL-C, CRP, glucose, and haemoglobin A1c (HbA1c), as well as missing records of covariates (*n* = 12,285). Finally, 13,726 participants in the NHANES and 3,964 participants in the CHARLS were eligible for this cross-sectional analysis. All participants supplied written informed consent in two large surveys, and two surveys were administered in conformity with the 1975 Helsinki Declaration.

### Exposure and outcome variables

In the fasting state, blood specimens were collected by professional medical workers and measured in the central laboratory. The TyG index was computed using the following formula: Ln (glucose [mg/dL]×triglycerides [TG] [mg/dL]/2) [22]. The formula for non-HDL-C was TC minus HDL-C. The main outcome of this study was arterial stiffness. Arterial stiffness was represented by ePWV. The ePWV was calculated using a coordinate Eqs. [23, 24]: mean blood pressure (MBP) was computed as DBP + 0.4×(SBP − DBP). ePWV = 9.587 – (0.402 × age) + (4.560 × 10^− 3^× age^2^) − (2.621 × 10^− 5^ × age^2^ × MBP) + (3.176 × 10^− 3^×age × MBP) − (1.832 × 10^− 2^× MBP). Increased arterial stiffness was defined as elevated ePWV, which was defined as a level higher than the 75th percentile of ePWV [25].

### Data collection

Information on demographic elements (including sex, age and race/ethnicity), health habits (history of drinking and smoking), health situations (diabetes and hypertension) and medical history (antihypertensive treatment and antidiabetic treatment) was acquired via face-to-face interviews and a standardized questionnaire.

The primary anthropometric indicators that were measured in this study were blood pressure (mm Hg), height (meter, m) and body weight (kilogram, kg). Body weight [kg]/height squared [m^2^] is the formula applied to calculate the body mass index (BMI). Individuals were asked to rest quietly for five minutes before blood pressure was measured; moreover, three measurements of DBP and SBP were made, and their mean values were documented.

Fasting blood samples were collected for tests of UA, TC, CRP, HDL-C, TG, LDL-C, glucose, and HbA1c.

### Statistical analysis

To get precise estimates that are typical of both the Chinese and U.S. populations, all of the analyses were computed using the proper sample weights. Normally distributed clinical data are presented as the mean ± standard deviation (SD), and nonnormally distributed data are described as the median and interquartile range (IQR). Quantitative variables are described as counts and percentages (%). As appropriate, to explore variations in baseline characteristics across groups, categorical and continuous variables were compared via Student’s t test, the Mann‒Whitney U test and the chi‒square test, respectively.

The TyG index and non-HDL-C and CRP levels were grouped into quartiles (Q1, Q2, Q3 and Q4). Weighted multiple logistic regression/linear models were used to measure odds ratios (ORs)/β coefficients and 95% confidence intervals (CIs) for the relationships of the TyG index, non-HDL cholesterol, and CRP with increased arterial stiffness, including fully adjusted model (Model 2) and an unadjusted model (Model 1). Alcohol consumption, smoking status, BMI, sex, age, UA, HbA1c, diabetes status, hypertension status, antihypertensive treatment, and antidiabetic treatment were adjusted for in Model 2. When considering that antihypertensive treatment affects SBP and DBP and thereby alters ePWV, sensitivity analyses were applied to verify the relationship between the TyG index and arterial stiffness after excluding patients treated with antihypertensive agents. The nonlinearity of the relationship between the four variables was estimated utilizing restricted cubic spline (RCS) regression. A *P* value < 0.05 indicated a nonlinear dose‒response association. The TyG index, CRP and non-HDL-C were classified into two groups based on the median value in the corresponding populations. Afterwards, individuals were classified into eight groups based on combined evaluation of the TyG index and CRP and non-HDL-C values. Then, more stringent clinical cut-off values for non-HDL-C (3.4 mmol/L) in the NHANES and cut-off values for non-HDL-C (4.9 mmol/L) in the CHARLS were employed to estimate the relationships of the discordant/concordant TyG index, CRP, and non-HDL-C groups with increased arterial stiffness for sensitivity analysis. The study performed subgroup analyses in terms of sex (male/female), age (</≥ 60 years), BMI (</≥ 30 kg/m^2^), HbA1c (</≥ 6.5%), and antihypertensive treatment (yes or no) and interaction tests to estimate underlying alterations.

Insulin resistance can influence arterial stiffness through a proinflammatory state and dyslipidaemia. Mediation analysis was applied to estimate whether this association was mediated by non-HDL-C and CRP. Bootstrap analysis was utilized to estimate the mediating impacts [26]. Four different preprogrammed routes were used, including indirect (routes 2, 3, and 4) and direct (route 1) mediation effects, and their β coefficients were assessed: Route 1, TyG index (exposure)→increased arterial stiffness (outcome); Route 2, TyG index (exposure)→non-HDL-C (mediator)→ increased arterial stiffness (outcome); Route 3, TyG index (exposure) → CRP (mediator) → increased arterial stiffness (outcome); Route 4, TyG index (exposure) → non-HDL-C (mediator) → CRP (mediator) →increased arterial stiffness (outcome). The significance threshold was set at *P* < 0.05. R statistical software (version 4.3.2) and IBM SPSS software (version 25) were utilized to conduct the data analysis.

## Results

### Patient characteristics

A total of 13,726 participants in the NHANES and 3964 participants in the CHARLS were included in the analysis. The characteristics of individuals from both surveys are displayed in Table 1. Compared with individuals without increased arterial stiffness, those with increased arterial stiffness had a greater prevalence of hypertension (NHANES, 59.88% vs. 17.26%; CHARLS, 41.33% vs. 6.32%), diabetes (NHANES, 18.25% vs. 5.13%; CHARLS, 79.33% vs. 25.84%), smoking (NHANES, 64.33% vs. 6.43%; CHARLS, 43.85% vs. 10.69%) and alcohol consumption (NHANES, 61.08% vs. 54.86%; CHARLS, 61.49% vs. 17.06%). Moreover, individuals with increased arterial stiffness suffered from higher levels of traditional risk factors, such as SBP, DBP, CRP, non-HDL-C, UA, TG, the TyG index, glucose, TC, ePWV, and HbA1c, than individuals without increased arterial stiffness in both large surveys.

**Table 1:** Baseline characteristics of populations in NHANES and CHARLS

Variables	NHANES			CHARLS		
	Increased arterial stiffness (*n* = 3420)	Non-increased arterial stiffness (*n* = 10,306)	*P*-value	Increased Arterial stiffness (*n* = 992)	Non-increased arterial stiffness (*n* = 2972)	*P*-value
Age, years	71.49 ± 8.31	35.45 ± 14.66	< 0.001	74.82 ± 6.69	61.44 ± 8.24	< 0.001
Sex, n (%)			0.016			< 0.001
Male	1724(50.41%)	4975(48.27%)		684(68.95%)	2294(77.18%)	
Female	1696(49.59%)	5331(51.73%)		308(31.05%)	678(22.82%)	
Race/Ethnicity			< 0.001			
Non-Hispanic white	2016(58.95%)	4434(43.02%)				
Non-Hispanic black	567(16.58%)	2181(21.16%)				
Hispanic	750(21.93%)	3225(31.29%)				
Other race	87(2.54%)	466(4.53%)				
Smoking, n (%)			< 0.001			
Ever/current	2200(64.33%)	663(6.43%)		435(43.85%)	318(10.69%)	< 0.001
Never	1220(35.67%)	9643(93.57%)		557(56.15%)	2654(89.31%)	
Alcohol consumption, n (%)			< 0.001			< 0.001
Ever/current	2089(61.08%)	5654(54.86%)		610(61.49%)	507(17.06%)	
Never	1331(38.92%)	4652(45.14%)		382(38.51%)	2465(82.94%)	
Diabetes, n (%)			< 0.001			< 0.001
Yes	624(18.25%)	529(5.13%)		787(79.33%)	768(25.84%)	
No	2796(81.75%)	9777(94.87%)		205(20.67%)	2204(74.16%)	
Hypertension, n (%)			< 0.001			< 0.001
Yes	2048(59.88%)	1779(17.26%)		410(41.34%)	188(6.32%)	
No	1372(40.12%)	8527(82.74%)		582(58.66%)	2784(93.68%)	
SBP, mmHg	143.88 ± 22.06	116.30 ± 13.40	< 0.001	154.88 ± 21.63	132.69 ± 19.38	< 0.001
DBP, mmHg	70.51 ± 15.83	67.90 ± 12.54	< 0.001	83.43 ± 13.99	78.06 ± 12.16	< 0.001
BMI, Kg/m^2^	28.45 ± 5.68	27.75 ± 6.60	< 0.001	23.21 ± 3.54	23.59 ± 3.58	0.004
ePWV, m/s	12.04 ± 1.51	7.24 ± 1.07	< 0.001	13.27 ± 1.05	10.02 ± 1.42	< 0.001
CRP, mg/L	2.50(1.10,5.30)	1.70(0.60,4.20)	< 0.001	1.60(0.90,3.07)	1.20(0.60,2.30)	< 0.001
Glucose, mg/dL	113.43 ± 35.15	99.03 ± 26.67	< 0.001	104.05 ± 31.39	97.15 ± 26.71	< 0.001
HbA1c, %	5.90 ± 0.99	5.40 ± 0.83	< 0.001	6.16 ± 1.04	5.92 ± 0.87	< 0.001
TG, mmol/L	1.56 ± 0.74	1.34 ± 0.77	< 0.001	7.67 ± 4.46	6.45 ± 4.31	< 0.001
TC, mmol/L	5.22 ± 1.08	4.90 ± 1.07	< 0.001	10.66 ± 2.10	10.03 ± 1.98	< 0.001
HDL, mmol/L	1.43 ± 0.42	1.38 ± 0.41	< 0.001	2.90 ± 0.64	2.95 ± 0.64	0.062
LDL, mmol/L	3.07 ± 0.96	3.04 ± 0.91	0.236	5.08 ± 1.47	4.72 ± 1.32	< 0.001
Non-HDL-C, mmol/L	3.78 ± 1.04	3.52 ± 1.06	< 0.001	7.70 ± 1.98	7.07 ± 1.87	< 0.001
TyG index	7.23 ± 0.55	6.92 ± 0.60	< 0.001	8.71 ± 0.61	8.45 ± 0.60	< 0.001
UA, mg/dL	3.90 ± 0.98	3.53 ± 0.93	< 0.001	5.09 ± 1.48	4.73 ± 1.32	< 0.001
Antihypertensive treatment, n (%)	1892(55.32%)	1256(12.19%)	< 0.001	242(24.39%)	381(12.32%)	< 0.001
Antidiabetic treatment, n (%)	494(14.44%)	375(3.64%)	< 0.001	78(7.86%)	192(6.21%)	0.129

All values were presented as Mean ± SD, or counts (proportion)

### Logistic regression and restricted cubic spline analysis

**Fig. 1:**
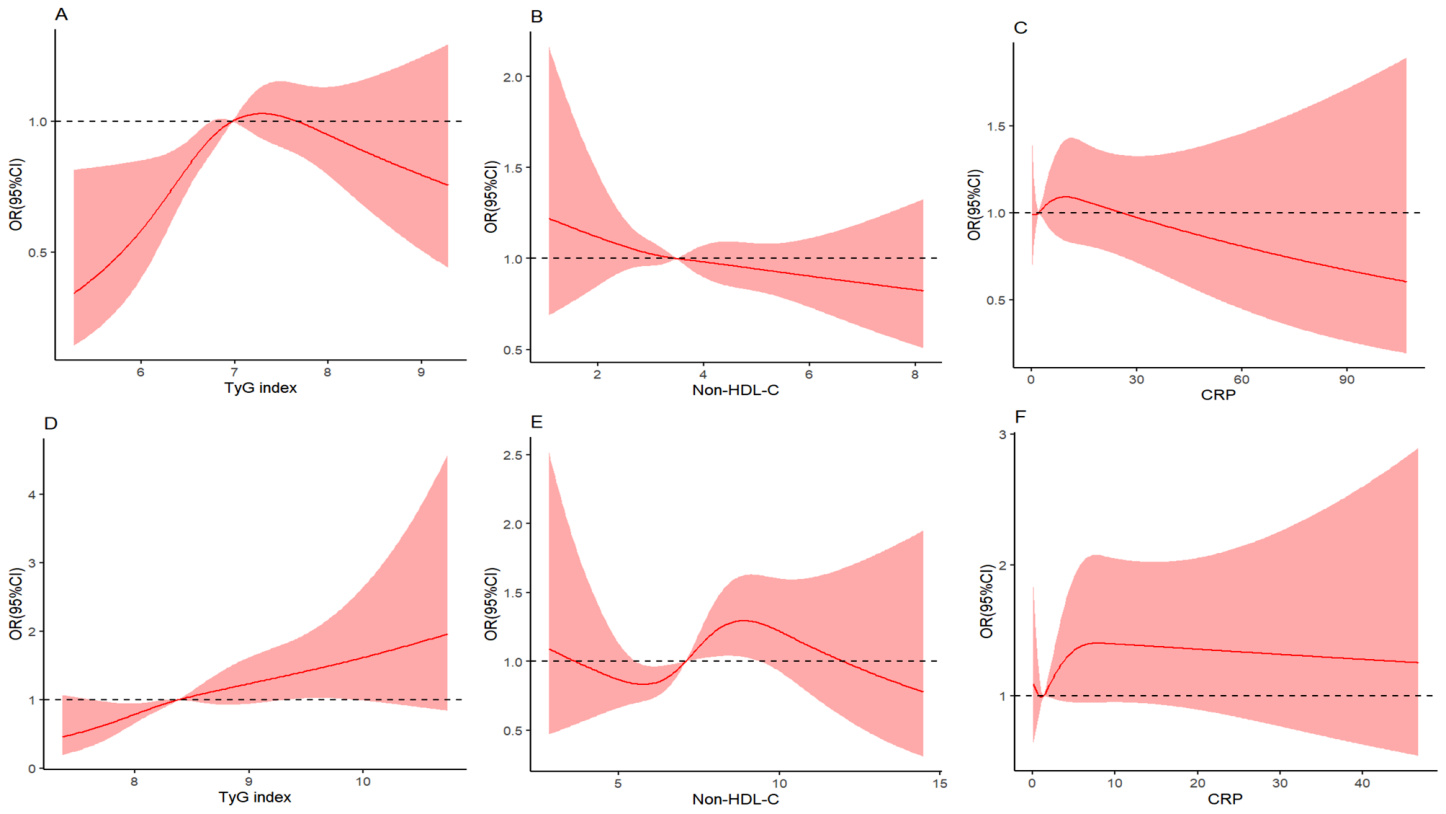
The restricted cubic spline curves of TyG index, non-HDL and CRP in the incidence of increased arterial stiffness. The RCS curves of TyG index (**A**), non-HDL (**B**) and CRP (**C**) in the incidence of increased arterial stiffness in NAHNES and the RCS curves of TyG index (**D**), non-HDL (**E**) and CRP (**F**) in the incidence of increased arterial stiffness in CHARLS. Adjusted for age, sex, BMI, UA, HbA1c, smoking, alcohol consumption, diabetes, hypertension, antihypertensive treatment, antidiabetic treatment

When the TyG index was used, non-HDL-C and CRP levels were considered categorical variables and divided into quartiles in the weighted logistic regression analysis. After full adjustment (Model 2), the ORs (95% CIs) for increased arterial stiffness in the higher quartiles (Q2-Q4) of the TyG index were 1.843 (1.444–2.352), 2.536 (1.994–3.210), and 2.610 (2.043–3.334) in the NHANES and 1.170 (0.785–1.745), 1.540 (1.053–2.251), and 1.579 (1.057–2.360) in the CHARLS, respectively, compared to the first quartile of the TyG index. Similarly, when non-HDL-C or CRP were considered independent exposure variables, elevated levels of non-HDL-C or CRP were associated with a greater incidence of increased arterial stiffness. After full adjustment (Model 2), the odds of increased arterial stiffness in the higher quartiles of non-HDL-C (Q2-Q4) ranged from 1.847 to 3.212 in the NHANES and from 1.090 to 1.476 in the CHARLS. Accordingly, the odds of increased arterial stiffness in the higher quartiles (Q2-Q4) of CRP ranged from 1.585 to 1.693 in the NHANES and from 1.049 to 1.446 in the CHARLS, as shown in Table 2. In addition, as displayed in Table 3, after excluding patients treated with antihypertensive agents, the TyG index was still closely correlated with increased arterial stiffness. Notably, after adjusting for BMI, sex, age, UA, HbA1c, alcohol consumption, smoking status, diabetes status, hypertension status, antihypertensive treatment status, and antidiabetic treatment status, the RCS curves indicated significant linear relationships between the TyG index, non-HDL-C level and CRP level and increased arterial stiffness in both large surveys (all *P* values for nonlinearity > 0.05), as shown in Fig. 1.

**Table 2:** Odds ratios of increased arterial stiffness by TyG index, non-HDL and CRP in the NHANES and the CHARLS

Exposure variables	NHANES		CHARLS	
	Increased arterial stiffness (OR 95%CI)		Increased arterial stiffness (OR 95%CI)	
	Model 1	Model 2	Model 1	Model 2
TyG index				
Q1	Reference	Reference	Reference	Reference
Q2	2.697(2.352–3.093) ***	1.843(1.444–2.352) ***	1.418(1.111–1.809) **	1.170(0.785–1.745)
Q3	3.933(3.444–4.490) ***	2.536(1.994–3.210) ***	3.239(2.586–4.051) ***	1.540(1.053–2.251) *
Q4	5.020(4.402–5.725) ***	2.610(2.043–3.334) ***	3.398(2.714–4.254) ***	1.579(1.057–2.360) *
Non-HDL-C				
Q1	Reference	Reference	Reference	Reference
Q2	1.398(1.243–1.578) ***	1.847(1.463–2.332) ***	1.107(0.886–1.383)	0.802(0.585–1.099)
Q3	1.783(1.590–1.999) ***	2.588(2.063–3.247) ***	1.579(1.277–1.952) ***	1.090(0.803–1.480)
Q4	2.011(1.795–2.252) ***	3.212(2.566–4.022) ***	2.227(1.812–2.737) ***	1.476(1.090–2.001) *
CRP				
Q1	Reference	Reference	Reference	Reference
Q2	2.213(1.963–2.496) ***	1.585(1.273–1.973) ***	1.442(1.154–2.114) **	1.049(0.762–1.446)
Q3	2.591(2.301–2.917) ***	1.697(1.345–2.140) ***	1.714(1.381–2.127) ***	1.049(0.764–1.439)
Q4	2.550(2.265–2.870) ***	1.693(1.249–2.573) ***	2.906(1.944–2.978) ***	1.466(1.066–2.017) *

Model 1: Unadjusted

Model 2: Adjusted for age, sex, BMI, UA, HbA1c, smoking, alcohol consumption, diabetes, hypertension, antihypertensive treatment, antidiabetic treatment. *p < 0.05; **p < 0.01; ***p < 0.001

**Table 3:** Odds ratios of increased arterial stiffness by TyG index in people without antihypertensive treatment

Exposure variables	NHANES		CHARLS	
	Increased arterial stiffness (OR 95%CI)		Increased arterial stiffness (OR 95%CI)	
	Model 1	Model 2	Model 1	Model 2
TyG index				
Q1	Reference	Reference	Reference	Reference
Q2	2.653(2.259–3.115) ***	2.013(1.656–2.446) ***	1.111(0.847–1.457)	1.219(0.794–1.873)
Q3	4.308(3.684–5.031) ***	2.855(2.360–3.453) ***	3.428(2.696–4.359) ***	1.866(1.265–2.754) **
Q4	6.124(5.257–7.135) ***	3.463(2.852–4.204) ***	3.840(3.024–4.877) ***	2.029(1.348–3.054) **

Model 1: Unadjusted

Model 2: Adjusted for age, sex, BMI, UA, HbA1c, smoking, alcohol consumption, diabetes, hypertension, antidiabetic treatment. *p < 0.05; **p < 0.01; ***p < 0.001

### Linear regression and subgroup analysis

**Fig. 2:**
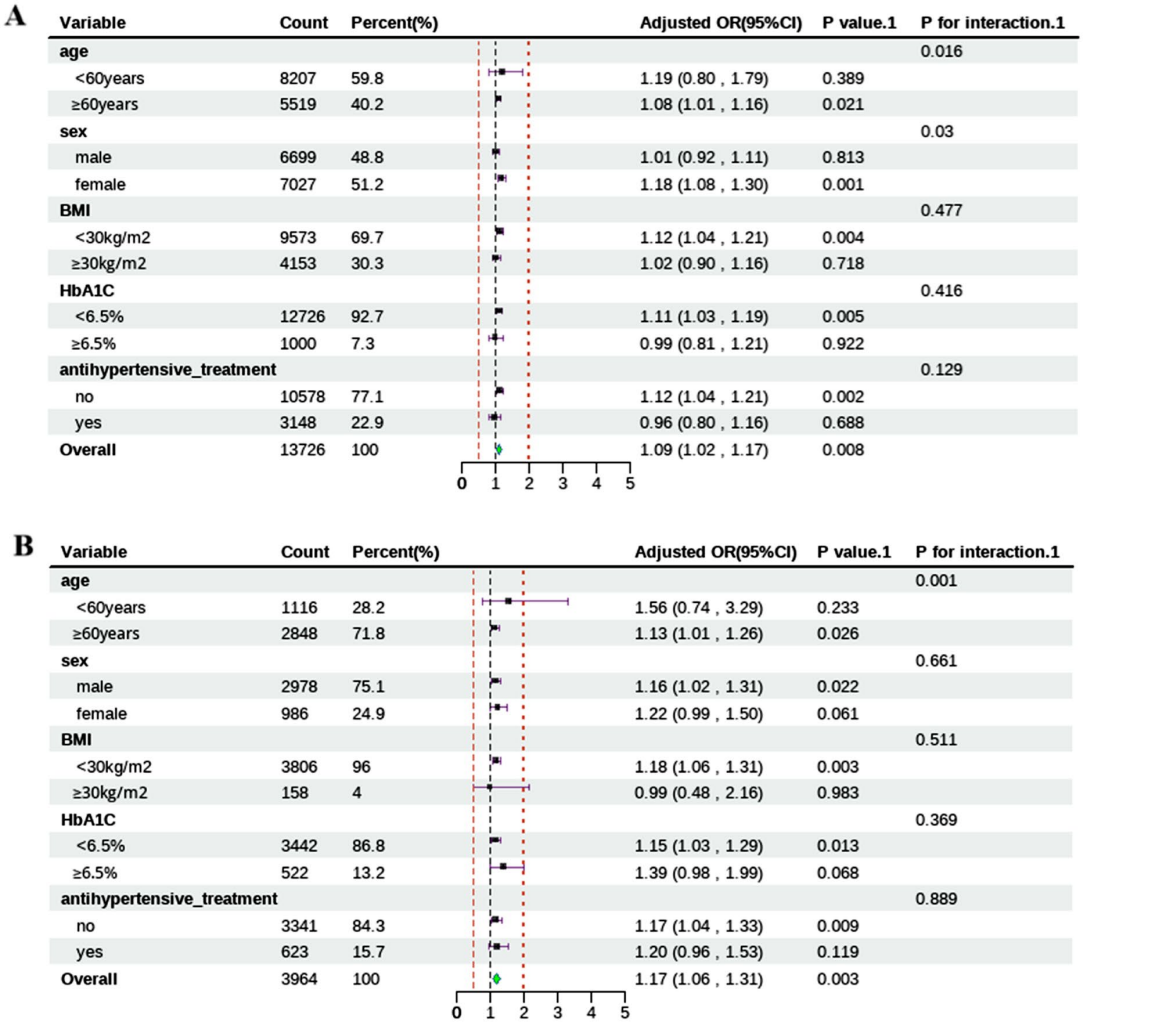
Subgroup analyses for the association of TyG index with increased arterial stiffness in NHANES (**A**) and CHARLS (**B**). Adjusted for age, sex, BMI, UA, HbA1c, smoking, alcohol consumption, diabetes, hypertension, antihypertensive treatment, antidiabetic treatment

As shown in Table 4, linear regression analyses confirmed that after fully adjustment (Model 2), the TyG index, non-HDL-C and CRP were still substantially connected to ePWV in both surveys. Both U.S. and Chinese participants with stronger insulin resistance (as evaluated by the TyG index), worsened lipid profiles (as estimated by non-HDL-C levels), and greater inflammation status (as assessed by CRP levels) demonstrated a worse status of arterial stiffness than did the reference groups. As presented in Fig. 2, in the subgroup analysis stratified by sex (male/female), age (</≥ 60 years), HbA1c (</≥ 6.5%), antihypertensive treatment (yes or no) and BMI (</≥ 30 kg/m^2^), sex and age were identified as being the interactive factors influencing correlation between TyG index and increased arterial stiffness in the NHANES (both *P* values for interaction < 0.05). Additionally, age was considered to be a marked interaction factor (*P* for interaction < 0.05) in the CHARLS.

**Table 4:** Beta between ePWV by TyG index, non-HDL and CRP in the NHANES and the CHARLS

Exposure variables	NHANES		CHARLS	
	ePWV (β 95%CI)		ePWV (β 95%CI)	
	Model 1	Model 2	Model 1	Model 2
TyG index				
Q1	Reference	Reference	Reference	Reference
Q2	0.969(0.861–1.078) ***	0.347(0.282–0.412) ***	0.418(0.266–0.569) ***	0.249(0.141–0.357) ***
Q3	1.471(1.363–1.579) ***	0.513(0.446–0.579) ***	1.979(1.827–2.131) ***	1.107(0.985–1.229) ***
Q4	1.865(1.756–1.973) ***	0.571(0.501–0.642) ***	2.013(1.862–2.165) ***	1.182(1.069–1.295) ***
Non-HDL-C				
Q1	Reference	Reference	Reference	Reference
Q2	0.499(0.388–0.610) ***	0.321(0.258–0.384) ***	0.431(0.265–0.596) ***	0.207(0.093–0.321) ***
Q3	0.917(0.806–1.029) ***	0.579(0.515–0.644) ***	0.984(0.819–1.149) ***	0.558(0.443–0.673) ***
Q4	1.140(1.029–1.251) ***	0.777(0.712–0.843) ***	1.340(1.175–1.505) ***	0.648(0.530–0.765) ***
CRP				
Q1	Reference	Reference	Reference	Reference
Q2	0.906(0.795–1.016) ***	0.307(0.242–0.372) ***	0.714(0.547–0.881) ***	0.341(0.225–0.457) ***
Q3	1.042(0.932–1.151) ***	0.318(0.247–0.389) ***	1.038(0.873–1.202) ***	0.509(0.392–0.625) ***
Q4	1.138(1.028–1.249) ***	0.372(0.305–0.439) ***	1.336(1.169–1.503) ***	0.554(0.432–0.676) ***

Model 1: Unadjusted

Model 2: Adjusted for age, sex, BMI, UA, HbA1c, smoking, alcohol consumption, diabetes, hypertension, antihypertensive treatment, antidiabetic treatment. *p < 0.05; **p < 0.01; ***p < 0.001

### Relationships of the discordant/concordant TyG index, CRP level and non-HDL level with increased arterial stiffness

**Fig. 3:**
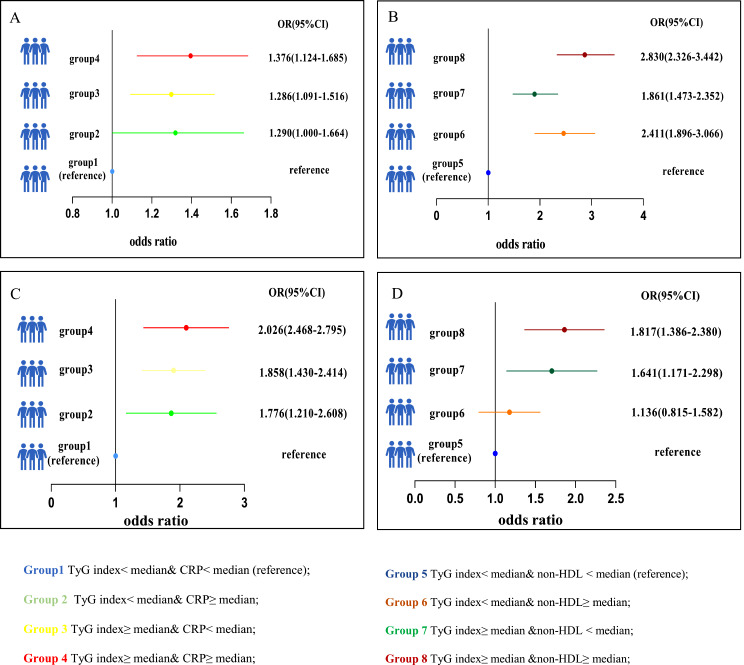
Association of discordance/concordance of TyG index, CRP and non-HDL-C (mean value) with increased arterial stiffness in NHANES (**A**–**B**) and in CHARLS (**C**–**D**). Adjusted for age, sex, BMI, UA, HbA1c, smoking, alcohol consumption, diabetes, hypertension, antihypertensive treatment, antidiabetic treatment

**Fig. 4:**
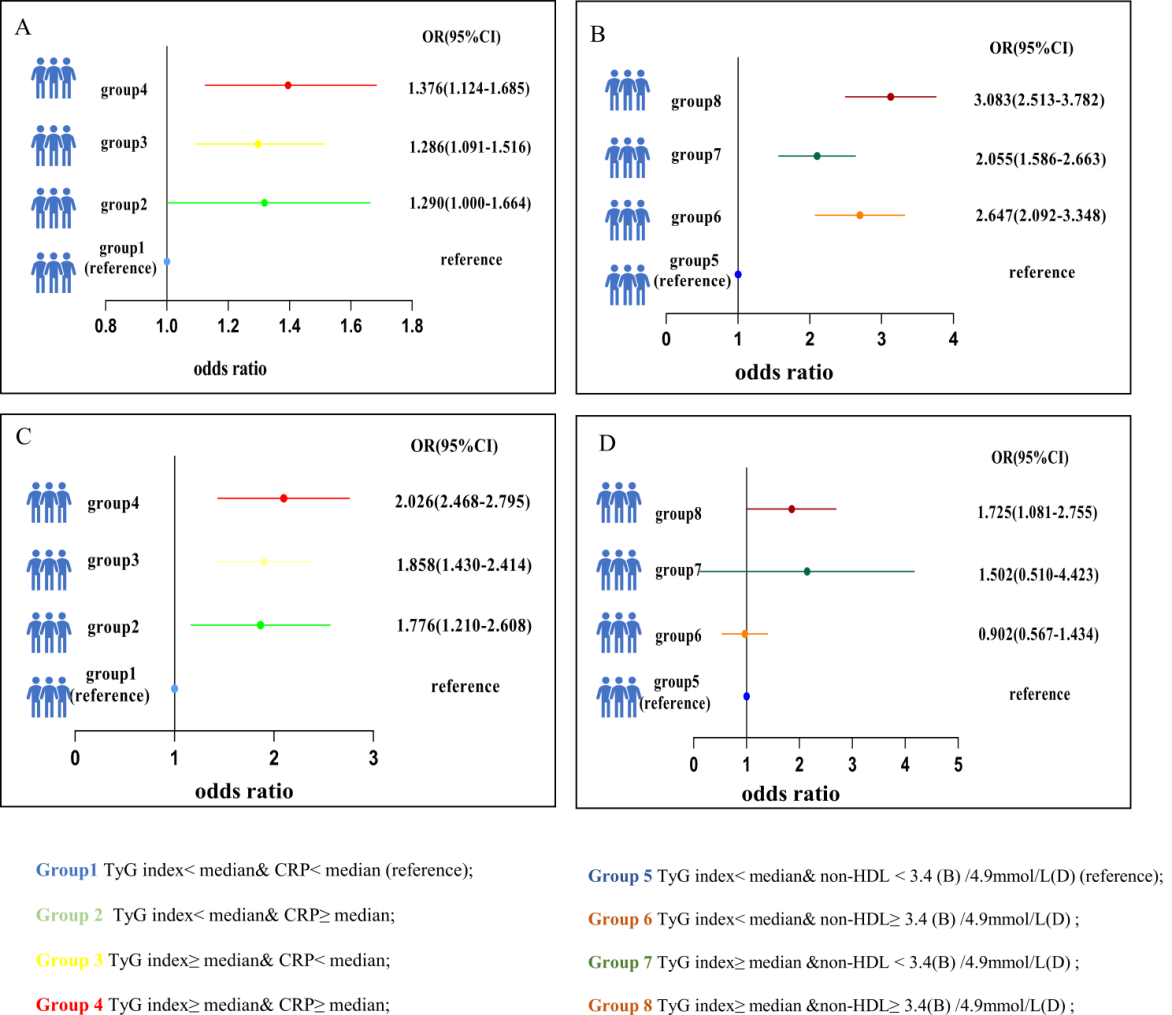
Association of discordance/concordance of TyG index, CRP and non-HDL-C (3.4mmol/L/4.9mmol/L-cutoffs) with increased arterial stiffness in NHANES(**A**–**B**) and in CHARLS (**C**–**D**). Adjusted for age, sex, BMI, UA, HbA1c, smoking, alcohol consumption, diabetes, hypertension, antihypertensive treatment, antidiabetic treatment

As demonstrated in Fig. 3; Table 5, using the multivariate adjustment model (Model 2), discordant/concordant TyG index and CRP levels or discordant/concordant TyG index and non-HDL-C levels were positively linked to increased arterial stiffness. Compared with individuals with lower TyG index (less than median value)/lower CRP level (less than median value) or lower TyG index (less than median value)/lower non-HDL-C level (less than median value) in the NHANES, people with higher TyG index/higher CRP level had the highest prevalence of increased arterial stiffness (OR = 1.376; 95% CI = 1.124–1.685). Likewise, people with higher TyG index/higher non-HDL-C level also had the highest incidence of increased arterial stiffness (OR = 2.830; 95% CI = 2.326–3.442). Similarly, the same analysis was repeated in the CHARLS dataset; specifically, individuals with a higher TyG index/higher CRP level (OR = 2.026; 95% CI = 1.468–2.795) or a higher TyG index/higher non-HDL-C level (OR = 1.817; 95% CI = 1.386–2.380) had the highest incidence of increased arterial stiffness. As summarized in Fig. 4, the results remained consistent when the stricter clinical non-HDL-C cut-off point (3.4 mmol/L) in the NHANES and the cut-off point (4.9 mmol/L) in the CHARLS were utilized for the sensitivity analyses.

**Table 5:** Associations of the TyG index, CRP and non-HDL with the risk of increased arterial stiffness

	NHANES		CHARLS	
	OR (95%CI)	p-value	OR (95%CI)	p-value
TyG index < median	Reference		Reference	
TyG index ≥ median	1.825(1.556–2.139)	< 0.001	1.884(1.502–2.364)	< 0.001
CRP < median	Reference		Reference	
CRP ≥ median	2.072(1.785–2.406)	< 0.001	1.314(1.060–1.628)	0.013
Non-HDL < median	Reference		Reference	
Non-HDL ≥ median	1.184(1.047–1.338)	< 0.001	1.393(1.097–1.769)	0.006
TyG index < median& CRP < median (group1)	Reference		Reference	
TyG index < median& CRP ≥ median (group2)	1.290(1.000-1.664)	0.003	1.776(1.210–2.608)	0.003
TyG index ≥ median& CRP < median (group3)	1.286(1.091–1.516)	0.002	1.858(1.430–2.414)	< 0.001
TyG index ≥ median& CRP ≥ median (group4)	1.376(1.124–1.685)	< 0.001	2.026(1.468–2.795)	< 0.001
TyG index < median& non-HDL < median (group5)	Reference		Reference	
TyG index < median& non-HDL ≥ median (group6)	2.411(1.896–3.066)	< 0.001	1.136(0.815–1.582)	0.452
TyG index ≥ median& non-HDL < median (group7)	1.861(1.473–2.352)	< 0.001	1.641(1.171–2.298)	0.004
TyG index ≥ median& non-HDL ≥ median (group8)	2.830(2.326–3.442)	< 0.001	1.817(1.386–2.380)	< 0.001

Adjusted for age, sex, BMI, UA, HbA1c, smoking, alcohol consumption, diabetes, hypertension, antihypertensive treatment, antidiabetic treatment. *p < 0.05; **p < 0.01; ***p < 0.001

### Mediation analysis

**Fig. 5:**
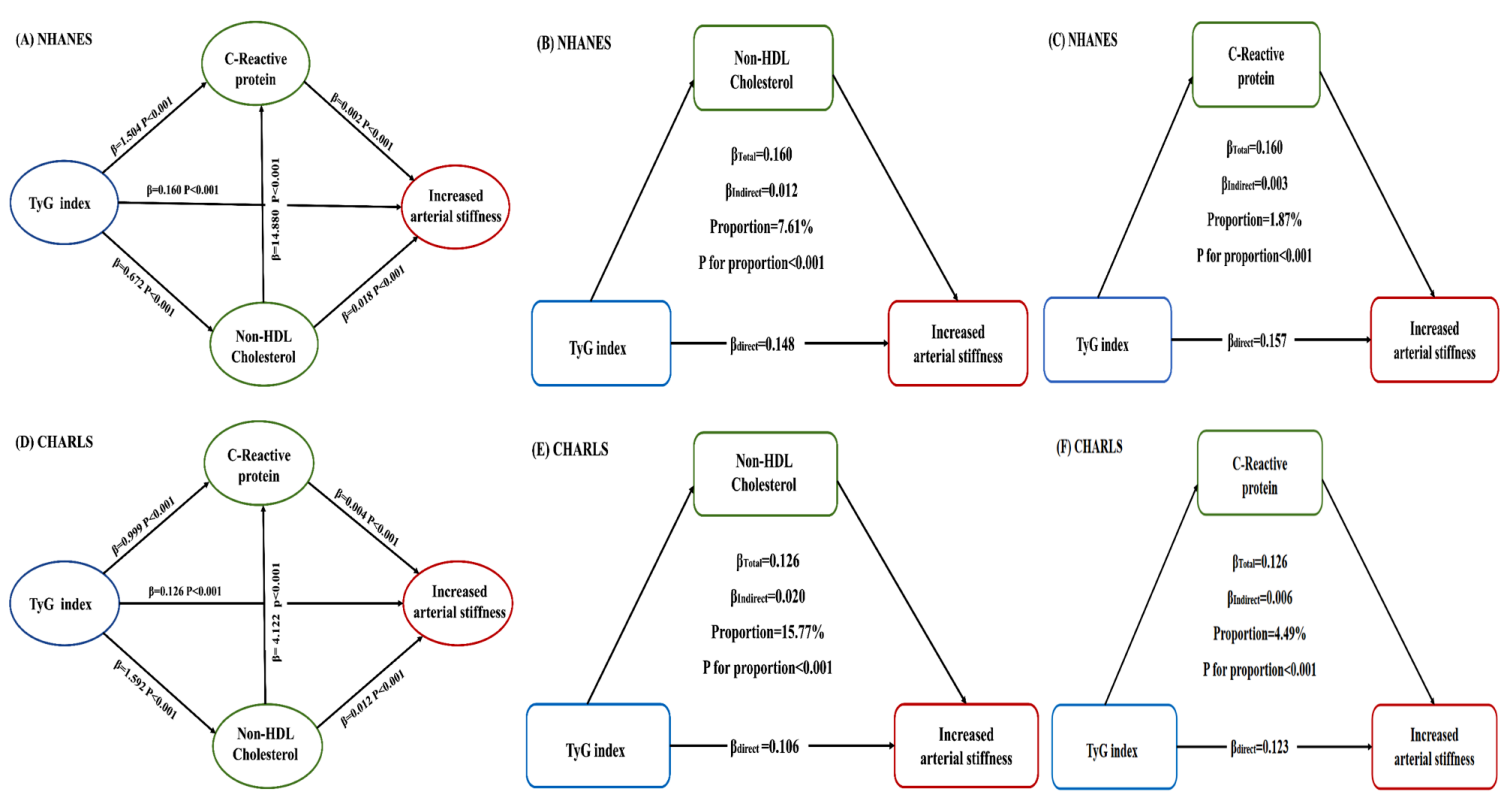
Mediation analysis of the relationship between TyG index and increased arterial stiffness. The graphs in (**A**–**C**) represented the mediating role of non-HDL and CRP in NHANES (unadjusted); The graphs in (**D**–**F**) represented the mediating role of non-HDL and CRP in CHARLS (unadjusted)

However, the degree to which the TyG index is correlated with arterial stiffness via lipids and inflammation remains largely unclear. This prompted us to investigate the mediating effects of non-HDL-C and CRP on this association. Figure 5 shown that this association was mediated by non-HDL-C (mediated effect: β = 0.012; *P* < 0.001) and CRP (mediated effect: β = 0.003; *P* < 0.001); these two indices explained 7.61% and 1.87%, respectively, of the relationship in the NHANES. More importantly, the mediating effect of Route 4 significantly explained 11.16% of the variance in the NHANES data. The same analysis was repeated in the CHARLS database, and both non-HDL-C (mediated effect: β = 0.020; *P* < 0.001) and CRP (mediated effect: β = 0.006; *P* < 0.001) accounted for 15.77% and 4.49%, respectively, of the relationship. Similarly, the mediating effect of Route 4 also significantly explained 18.75% of the association in the CHARLS. These data further support our hypothesis that lipid and inflammation biomarkers are key mediators of this association.

## Discussion

This study demonstrated that (1) after fully adjustment, the TyG index, non-HDL-C level and CRP level were strongly associated with arterial stiffness, which was represented by categorical and continuous measures in two large population-based surveys; (2) individuals with a higher TyG index had a 1.0–2.0 times greater chance in the U.S. population, and individuals in the Chinese population had a 1.5–1.6 times greater chance, of being diagnosed with arterial stiffness; (3) the highest risk of increased arterial stiffness was detected in both participants with higher TyG index/higher CRP level and those with higher TyG index/higher non-HDL-C level in two surveys; and (4) lipid levels (non-HDL-C) and inflammation status (CRP) were not only independently correlated with arterial stiffness but were also partially mediated by this association.

Numerous reports have shown a crucial linked between TyG index and arterial stiffness. For example, 3,185 patients with diabetes in a single-centre study shown that the TyG index was more positively linked to increased arterial stiffness than was conventional indicator of IR [13]. A recent study supported the idea that the TyG index also increased the risk of the progression of arterial stiffness [14].Additionally, the TyG index, which is an economically efficient marker, has been deemed as a predictor of CVD [10, 27]. Notably, the impacts of the TyG index on colon tumours, diabetes, heart failure, liver disease, and ischaemic stroke have also been reported [28–31].

Dyslipidaemia, especially hypercholesterolemia, can cause changes in vascular stiffness and increase the risk of CVD. Notably, non-HDL cholesterol included “bad cholesterol” [15]. Unsurprisingly, non-HDL-C was a better predictor of CVD than LDL cholesterol alone [17]. Additionally, non-HDL-C is closely associated with insulin resistance [32]. Indeed, an increasing amount of data indicate that increased non-HDL-C has the potential to be considered a marker for the early diagnosis of arterial stiffness [33]. Notably, insulin resistance is also a well-established contributor to inflammation. Moreover, many reports have indicated that high levels of inflammation synergically increase the risk for arterial stiffness and chronic coronary syndrome [6, 34]. Furthermore, increased inflammation, such as via increased C-reactive protein (CRP) levels, may be related to the progression of dyslipidaemia-induced arterial stiffness [20].

Given that lipid and inflammation levels have been verified as being remarkable risk markers for arterial stiffness, we speculate that lipid and inflammation levels could mediate the correlation of arterial stiffness with the TyG index. This study suggested that higher TyG index, CRP and non-HDL values were strongly related to a greater incidence of increased arterial stiffness. Importantly, the results further indicated that CRP and non-HDL indices both partially mediated the relationship between arterial stiffness and the TyG index. Moreover, the study also demonstrated that females are more sensitive to arterial stiffness from TyG index exposure than males according to the NHANES, which was consistent with data from previous reports [13, 35]. In addition, Su et al. [36] found a stronger influence of the TyG index on arterial stiffness among older Chinese people aged over 60 years, which was also observed in this study. The results also suggested a stronger influence of the TyG index on increased arterial stiffness in older people in the NHANES and CHARLS. We believe that these disparities may be associated with differences in participant selection. Notably, women and older people require stricter IR control, and further investigations are needed to estimate the age and sex differences in this correlation in other populations.

Some possible mechanisms could explain the complex relationships among the TyG index, lipid and inflammation levels, and arterial stiffness or vasculopathy. Metabolic syndrome is closely correlated with IR, which is represented by endothelial dysfunction (ED) and a proinflammatory state [37]; moreover, studies have reported that these phenomena play an important part in the sophisticated pathobiology of arterial stiffness [38]. Generally, IR can cause systemic metabolic disorders through hyperglycaemia, later dyslipidaemia and low-grade inflammation [39, 40]. Another reason for this effect may involve dyslipidaemia, which is a traditional risk marker for insulin resistance and arterial stiffness [41]. Dyslipidaemia is consistently involved in chronic inflammation-related pathologies such as diabetes and atherosclerosis [42, 43]. Importantly, increasing levels of inflammation may trigger dyslipidaemia-induced arterial stiffness. Moreover, high CRP levels could explain the involvement of arterial stiffness and inflammation. CRP is not only a marker that is linked to vascular inflammation and subclinical inflammatory states but could also cause unnatural physiological variations in arterial wall damage [44].

### Strengths and limitations

Until now, this is the first large-scale study to uncover that lipid levels and inflammation status were key mediators of this association in both large surveys, providing a new perspective for future studies exploring the underlying mechanisms of arterial stiffness. Additionally, the study also demonstrated a significant linear relationship between the four variables in general populations. However, this study had several limitations. First, although possible traditional risk markers were adjusted for, all of the confounding factors cannot be ruled out in the analysis. Second, it is unable to verify the causal correlations of lipid and inflammation levels or the TyG index with arterial stiffness due to the nature of cross-sectional study. Third, American and Chinese populations were included in this study, and the results may not be applied to other populations; thus, further research is necessary to verify these findings in other populations. Finally, the mediation analysis assumed a certain sequence of impacts; however, the directional resolution of these effects was restricted by the cross-sectional study design. Therefore, this is a general potential restriction that is experienced by cross-sectional studies.

## Conclusions

This study provided evidence for the correlation of the TyG index, non-HDL-C and CRP with arterial stiffness among general populations from two large population-based surveys. In addition, lipid and inflammation biomarkers could be key mediators linking TyG index with increased arterial stiffness. These findings have crucial clinical significance for identifying the pathogenic mechanism underlying insulin resistance, which induces damage to the vascular wall. In addition to IR, these findings also support the significance of reducing lipid and inflammation on arterial stiffness risk in general populations, which could provide a new preventive measure for reducing risk of arterial stiffness in clinical practice.

## Data Availability

The NHANES dataset in this study are openly available from the Centers for Disease Control and Prevention at https://www.cdc.gov/nchs/nhanes/index.htm. Data are available in a public, open access repository. The CHARLS datasets are available on request from their home page at http://charls.pku.edu.cn/.

## Declarations

### Authors’ information

^1^The First School of Clinical Medicine, Southern Medical University, Guangzhou, 510515 Guangdong China.^2^Department of Endocrinology, General Hospital of Central Theater Command, Wuhan, 430070 Hubei China. ^3^Shantou University Medical College, Shantou, Guangdong Province, China;

## Abbreviations

NHANES: National Health and Nutrition Examination Survey
CHARLS: China Health and Retirement Longitudinal Study
CRP: C-reactive protein
Non-HDL-C: Non-high-density lipoprotein cholesterol
BMI: Body mass index
HbA1c: Glycosylated hemoglobin A1c
ePWV: Estimated pulse wave velocity
RCS: Restricted cubic spline
HOMA-IR: Homeostasis model assessment of insulin resistance
